# Inhibition of Autophagy Prevents Panax Notoginseng Saponins (PNS) Protection on Cardiac Myocytes Against Endoplasmic Reticulum (ER) Stress-Induced Mitochondrial Injury, Ca^2+^ Homeostasis and Associated Apoptosis

**DOI:** 10.3389/fphar.2021.620812

**Published:** 2021-03-08

**Authors:** Jun Chen, Li Li, Xueyang Bai, Lili Xiao, Jiahong Shangguan, Wenjing Zhang, Xiangqin Zhang, Shen Wang, Gangqiong Liu

**Affiliations:** Vasculocardiology Department, the First Affiliated Hospital of Zhengzhou University, Zhengzhou, China

**Keywords:** PNS, ER stress, autophagy, mitochondrial injury, ROS, RyR_2_ oxidation, apoptosis, Ca^2+^ homeostasis

## Abstract

Endoplasmic reticulum (ER) stress is often closely linked to autophagy, hypoxia signaling, mitochondrial biogenesis and reactive oxygen species (ROS) responses. Understanding the interaction between ER stress, mitochondrial function and autophagy is of great importance to provide new mechanisms for the pathology, prevention and treatment of cardiovascular diseases. Our previous study has reported that Panax notoginseng saponins (PNS) protection against thapsigargin (TG)-induced ER stress response and associated cell apoptosis in cardiac myocytes is calcium dependent and mediated by ER Ca^2+^ release through RyR_2_. However, whether its protection upon ER stress and associated apoptosis is related to mitochondrial function and autophagy remains largely unknown. Here, we investigated the roles of PNS played in TG-induced mitochondrial function, ROS accumulation and autophagy. We also assessed its effects on Ca^2+^ homeostasis, ER stress response and associated cell death in the presence of autophagy inhibition. PNS-pretreated primary cultured neonatal rat cardiomyocytes were stimulated with TG to induce ER stress response. Mitochondrial potential (Δ*ψ*m) was measured by JC-1. The general and mitochondrial ROS were measured by DCFH-DA and MitoSOX Red, respectively. Autophagy was evaluated by immunofluorescence of LC3, and immunoblots of LC3, p62, ATG7 and PINK1. In addition, mRFP-GFP-LC3 labeling was used to assess the autophagic influx. SiATG7 transfected H9c2 cells were generated to inhibit autophagy. Cytosolic and ER Ca^2+^ dynamics were investigated by calcium imaging. RyR_2_ oxidation was tested by oxyblot. Cell viability was examined by TUNEL assay. ER stress response and cell apoptosis were detected by immunoblots of BiP, CHOP, Cleaved Caspase-3 and Caspase-12. The results demonstrated that firstly, PNS protects against TG-induced mitochondrial injury and ROS accumulation. Secondly, PNS enhances autophagy in TG-induced cardiac myocytes. Thirdly, inhibition of autophagy diminishes PNS prevention of TG-induced mitochondrial injury, ROS accumulation and disruption of Ca^2+^ homeostasis. Last but not least, inhibition of autophagy abolishes PNS protection against TG-induced ER stress response and associated apoptosis. In summary, PNS protection against ER stress response and associated apoptosis is related to the regulation of mitochondrial injury and ROS overproduction via modulation of autophagy. These data provide new insights for molecular mechanisms of PNS as a potential preventive approach to the management of cardiovascular diseases.

## Introduction

The endoplasmic reticulum (ER) is a subcellular organelle that is essential to the intracellular protein synthesis, folding, and structural maturation. It is predominantly known as a protein-folding checkpoint, which serves to ensure the proper folding and maturation of newly secreted proteins and transmembrane proteins (Anelli and Sitia, 2008). Many pathological conditions, such as ATP perturbation, calcium fluctuation, hypoxia, infection, inflammation and nutrient deprivation, contribute to ER dyshomeostasis, thus leading to a reduction of the protein-folding potential and eventually initiating the accumulation and aggregation of unfolded proteins in the ER lumen, acknowledged as the ER stress (Merksamer et al., 2008). The ER stress enacts the unfolded protein response (UPR) as an adaptive response for maintaining protein homeostasis (Kozutsumi et al., 1988). However, UPR caused by severe ER stress situation is unable to maintain the homeostasis in the ER and thus activates intrinsic apoptosis, and cells finally undergo apoptosis process (Szegezdi et al., 2006; Merksamer and Papa, 2010).

Autophagy is a cellular self-digestion process that plays critical roles in physiological processes such as cell growth, cell cycle and differentiation, as well as programmed cell death including apoptosis and senescence by the lysosomal degradation and the exclusion of perennial and misfolded proteins, pernicious cellular substances, pernicious organelles and infecting pathogens, as well as the maintenance of the energy homeostasis (Yang and Klionsky, 2010; Settembre and Ballabio, 2014). Autophagy protects against neurodegenerative disorders, infections, inflammatory diseases and cancer (Dutta et al., 2013). Indeed, autophagy is also essential for the normal maintenance, repair and adaptation of the heart due to perinatal starvation or various stresses (Kostin et al., 2003). Additionally, mitochondria-induced oxidative stress and consequent reactive oxygen species (ROS) overproduction may further disturb cardiac function and autophagy can selectively remove damaged mitochondria and preserve heart function (Dutta et al., 2013).

Although ER stress and autophagy can function independently, they share a number of common features including relieving cell stress and inducing cell death under extreme conditions (Hayashi-Nishino et al., 2009). Upon UPR stimulation, autophagy mainly functions to protect against cell death. However, under certain circumstances autophagy might be required to activate the cell death machinery (Rashid et al., 2015). Nevertheless, the relationship between autophagy and ER stress is not yet fully understood.

Panax notoginseng (Sanqi), a traditional Chinese medicinal herb known as a hemostatic medicine, has been used to accelerate blood clotting, alleviate swelling and relieve pain for more than a thousand years (Lee et al., 2012). Panax notoginseng saponin (PNS) extracted from Panax notoginseng is commonly used for treatment of cardiovascular disorders. Our previous study has shown that PNS protects against thapsigargin (TG)-induced ER stress response and its associated cell apoptosis in cardiac myocytes through the regulation of ER calcium release mediated by RyR_2_ (Chen et al., 2019). There is an evidence showing that PNS treatment enhanced HIF-1*α*, which in turn increased autophagy in renal tissue (Liang et al., 2017). Its main component, such as Ginsenosides-Rb1, protects neuron structure by recovering the autophagy genes LC3 and Beclin1 suggesting that autophagy may have a role in PNS protection of cell death (Liu et al., 2011).

The current work has attempted to integrate the autophagy and ER stress pathways, and provide a better understanding of a new mechanism of PNS protection of cardiac myocytes apoptosis toward ER stress through mitochondria oxidation and autophagy pathways. Therefore, we first examined the effects of PNS on TG-induced mitochondria morphology, mitochondrial membrane potential (Δ*ψ*m), ROS accumulation, as well as cell autophagy. We then investigated the effects of PNS on TG-induced mitochondrial injury, ROS production, intracellular Ca^2+^ homeostasis, as well as the ER stress response and associated cell death with the inhibition of autophagy. These findings thus identified a crosstalk between ER stress and autophagy pathways that involved in a new mechanism of PNS promotion of cell survival toward intracellular stress.

## Materials and Methods

### Chemicals and Treatment

PNS was purchased from Kunming Pharmaceutical Corporation (Yunnan, China; patent no. ZL96101652.3) with the major effective constituents including notoginsenoside R1 9.8% (v/v), ginsenoside Rb1 32.3% (v/v), ginsenoside Rg1 35.3% (v/v), ginsenoside Re 4.0% (v/v), and ginsenoside Rd 4.9% (v/v) and the total pharmaceutical concentration of 90% (v/v). TG (content ≥98%) was purchased from Sigma (T9033, Sigma-Aldrich, United States). High performance liquid chromatograph (HPLC) of the main PNS components has shown in the previous report (Chen et al., 2019). For drug treatment, cells were pretreated with 40 *μ*g/ml PNS for 12 h and then added 1 μm TG for 12 h.

### Primary Culture of Neonatal Rat Cardiomyocytes and Purification Identification

Cardiomyocytes were obtained by dissociating hearts of neonatal Sprague–Dawley rats (1–3 days old). The experimental protocol for animals was approved by the Ethics Committee for Scientific Research and Clinical Trials of the Affiliated Hospital of Zhengzhou University. The detailed isolation and purification of cardiomyocyte was referred to the previous protocols (Chen et al., 2019). Briefly, cardiomyocytes were isolated using a neonatal cardiomyocytes isolation system (LK003303, Worthington Biochemical, United States). After the fibroblasts and endothelial cells were extracted, the cardiomyocytes were then seeded onto cell culture plates pre-coated with 10 μg/ml of fibronectin and cultured in DMEM-F12 media containing 10 mM HEPES, 10% FBS, penicillin–streptomycin (100 U/ml–100 μg/ml), and 0.1 μmol/ml BrdU to inhibit non-myocyte cell proliferation. The cardiomyocytes were treated with PNS or TG after 72 h of tissue culture as indicated (Chen et al., 2019).

### Cell Culture and siRNA Transfection

Rat cardiomyoblast H9c2 cell line was obtained from the American Type Culture Collection (ATCC# CRL-1446TM, ATCC, United States). H9c2 cells were grown in DMEM containing 10% FBS, supplemented with l-glutamine (2 mm), penicillin (100 U/ml) and streptomycin (100 μg/ml), in a humidified atmosphere containing 5% CO_2_ for 24 h and then transfected with small interfering RNA (siRNA) against ATG7 (siATG7) (s161900, Thermo Fisher Scientific, United States) or with a select siRNA negative control (siNC) (4390843, Thermo Fisher Scientific, United States) using Attractene Transfection Reagent (301005, Qiagen, United States) following the instructions (Staskiewicz et al., 2013). The media was replaced after 24 h incubation with fresh media and then cultured for another 24 h before treatment of PNS or TG as indicated.

### Immunofluorescence Assays

For immunofluorescence assays, cells were fixed with 4% paraformaldehyde (PFA) and permeabilized with 0.2% Triton X-100. After blocking with 1% bovine serum albumin (BSA), cells were then incubated with the primary anti-calnexin antibody (AF18, 1:500, Thermo Fisher Scientific, United States), anti-TOM20 antibody (sc-17764, 1:500, Santa Cruz Biotechnology Inc., United States), anti-microtubule-associated protein 1A/1 B-light chain 3 (LC3B) (ab192890, 1:500, Abcam, United States) for 60 min at 37°C, respectively, followed by the corresponding secondary antibodies, Alexa 488 immunoglobulin G (IgG) (A32723, 1:1,000) or Alexa 568 IgG (A11011, 1:1,000) (Thermo Fisher, United States) in a dark chamber. Cells were examined using a laser scanning confocal microscope (LSM510, Carl Zeiss, Germany). Fluorescent images were acquired by a Plan-Neofluar 20×/0.40 LD or Plan-Apochromat 63×/1.40 Oil objective with either 488 nm laser excitation (525/50 nm band pass emission) or 543 nm laser excitation (570 nm long pass emission).

### Mitochondrial Morphology Score

80–100 random TOM20-labelled fluorescent mitochondria in cardiomyocytes were selected and scored as tubular, intermediate or fragmented by an investigator-blinded analysis. The network was scored as described in the previous report (Wiersma et al., 2019). Briefly, the network was scored as tubular when it appeared as long and intertwining tubules; as intermediate when the tubules were at least 30% shorter and showed dots; and as fragmented when >70% of the network showed dots instead of tubules. The number of cardiomyocytes showing tubular, intermediate or fragmented mitochondria was expressed as the percentage of the total cardiomyocytes.

### Mitochondrial Membrane Potential (Δ*ψ*m) Measurement

Mitochondrial membrane potential (Δ*ψ*m) in cardiomyocytes was assessed using 5, 5′, 6, 6′-tetrachloro-1, 1′, 3, 3′-tetraethylbenzimidazolyl-carbocyanine iodide (JC-1) (ab141387, Abcam, United States). In details, cardiomyocytes were seeded in a 96-well black frame plate at the density of 10,000 cells/well. Cells were exposed to 5 μ*m* JC-1 for 30 min and analyzed using a multi-label plate reader (VICTOR, PerkinElmer, United States) with 488 nm excitation; 530 nm emission for green and 590 nm emission for red, respectively. Alteration in the ionic equilibrium results in the mitochondria depolarization. Mitochondrial membrane potential was indicated by the ratio of the aggregated JC-1 (red fluorescence) and the monomeric JC-1 (green fluorescence).

### Western Blot Analyses

Protein (100–120 μg of total protein per lane) was separated through sodium dodecyl sulfate-polyacrylamide gel electrophoresis (SDS-PAGE) gels of 5% for ryanodine receptor 2 (RyR_2_); 10% for dynamin-related protein 1 (DRP1), mitofusin 2 (MFN2), phosphatase and tensin homolog (PTEN) induced kinase 1 (PINK1), sequestosome 1 (SQSTM1/P62), autophagy-related gene 7 (ATG7), or immunoglobulin heavy chain-binding protein (BiP); or 12% for LC3B, C/EBP homologous protein (CHOP), Cleaved Caspase-3, Caspase-12 or *β*-actin, and then transferred to polyvinylidene difluoride (PVDF) membranes. The PVDF membranes were probed with anti-MFN2 (#702768, 1:1,000, Invitrogen, United States), anti-DRP1 (ab184247, 1:1,000, Abcam, United States), anti-LC3B (ab192890, 1:500, Abcam, United States), anti-PINK1 (ab23707, 1:500, Abcam, United States), anti-SQSTM1/p62 (ab240635, 1:5,000, Abcam, United States), anti-ATG7 (ab133528, 1:3,000, Abcam, United States), anti-RyR_2_ (ab2868, 1:500, Abcam, United States), anti-BiP (ab21685, 1:1,000, Abcam, United States), anti-CHOP (sc-7351, 1:500, Santa Cruz Biotechnology Inc., United States), anti-Cleaved Caspase-3 (#9664, 1:800, Cell Signaling Technology, United States), or anti-Caspase-12 (#PA5-19963, 1:500, Invitrogen, United States) followed by appropriate horseradish peroxidase (HRP)-conjugated secondary antibodies. The *β*-actin (A5316, 1:10,000, Sigma-Aldrich, United States) gene was used as the internal standard for normalization of the protein samples. Chemoluminescence was revealed using Pierce™ Enhanced Chemoluminescence (ECL) Western Blotting Substrate (32106, Thermo Fisher Scientific, United States) and densitometry was performed using Quantity One 1-D software (Bio-Rad Laboratories, United States).

### Intracellular Reactive Oxygen Species (ROS) Measurement

Intracellular general ROS level was assessed by the fluorescent probe dichloro-dihydro-fluorescein diacetate (DCFH-DA) (ab113851, Abcam, United States). Cells were incubated with 20 μm DCFH-DA at 37°C for 30 min in dark. The fluorescent images of DCFH-DA staining were acquired by using a laser scanning confocal microscopy at an excitation wavelength of 488 nm and an emission wavelength of 525 nm (LSM510, Carl Zeiss, Germany). The average fluorescence intensity was analyzed by using an image analysis system to identify the general ROS production (Image J, National Institutes of Health, United States).

Additionally, mitochondrial superoxide production was evaluated by MitoSOX™ Red (M36008, Invitrogen/Molecular Probes, United States). Cells were loaded with 5 μm MitoSOX™ Red at 37°C for 30 min in dark, and were imaged by a laser scanning confocal at an excitation wavelength of 510 nm and an emission wavelength of 580 nm (LSM510, Carl Zeiss, Germany). MitoSOX Red fluorescence was analyzed by an image analysis system to identify the mean mitochondrial superoxide production (Image J, National Institutes of Health, United States).

### Measurements of Autophagic Flux

Autophagic flux was measured using the mRFP-GFP-LC3 reporter plasmid (21074, 1 μl/ml, Addgene, United States). Cardiomyocytes were transfected with the plasmid using lipofectamine 2000 (11668019, Invitrogen/Molecular Probes, United States) for 24 h and then imaged by a laser scanning confocal using a 488 nm laser excitation and emission was collected from 500 to 560 nm for GFP, and a 543 nm laser excitation and emission was collected at 600–660 nm for RFP (LSM510 Meta, Carl Zeiss, Germany). The formation of autophagosomes was observed as yellow puncta, and autolysosomes appeared as red only puncta in the merged images. Autophagic flux was determined based on the increase in the percentage of red only spots in the merged images.

### Cytosolic Ca^2+^ Measurements

Cells were plated in glass bottom 35 mm-petri dishes for 24 h (No. 1.5, MatTeK Corporation, United States). Intracellular Ca^2+^ labeling and imaging was described as before (Chen et al., 2019). In details, cells were labeled with 5 μm Fura-2 AM (F1221, Thermo Fisher Scientific, United States) supplied with 0.02% Pluronic F-127 (P3000MP, Thermo Fisher Scientific, United States) and then were illuminated at an alternating excitation wavelengths of 340 nm and 380 nm in an Epi-fluorescence microscope with a Plan-Fluor 40×/1.3 Oil objective (Eclipse Ti, Nikon, Japan). The emitted fluorescence was recorded at 510 nm with an Andor Zyla sCMOS camera (Oxford Instruments, United Kingdom). Exposure time was typically 100–200 ms, and images were collected every 10–20 s. Images were analyzed using MetaFluor software (Universal Imaging Corporation, United States). Fluorescent images were background corrected and cells with similar fluorescence intensity were analyzed.

### ER Ca^2+^ Measurements

Cells were plated in glass bottom 35 mm-petri dishes for 24 h (No. 1.5, MatTeK Corporation, United States) and transiently transfected with the fluorescence resonance energy transfer (FRET)-based D1ER cameleon (Palmer et al., 2004). FRET imaging was described as in the previous report (Chen et al., 2019). Briefly, cells were imaged by an Epi-fluorescence microscope with a Plan-Fluor 40×/1.3 Oil objective (Eclipse Ti, Nikon, Japan). The emission ratio of the cameleon was accomplished by 425 nm excitation wavelength with a dichroic mirror 515 nm and two emission filters (475 nm for ECFP and 535 nm for citrine-YFP) (Chroma Technology Corporation, United States). Changes in ER Ca^2+^ were expressed as the FRET-to-CFP emission ratio. Images were analyzed using MetaFluor software (Universal Imaging Corporation, United States).

### Oxidation of RyR_2_

Oxidation level of RyR_2_ was detected by reaction with 2, 4-dinitrophenyl hydrazine (DNP) using an OxyBlot™ Protein Oxidation Detection Kit (S7150, Millipore, United States) (Wang et al., 2015). Briefly, samples were denatured by SDS and the carbonyl groups in the protein side chains were derivatized to DNP-hydrazone by reaction with DNPH. The proteins were electrophoresed on a SDS-PAGE gel and followed by immunoblotting of the anti-DNP antibody (1:150). The same membrane was incubated with the anti-RyR_2_ antibody (1:500) for reblotting.

### Terminal Deoxynucleotidyl Transferase dUTP Nick End Labeling (TUNEL) Assay

The cardiac myocytes apoptosis was measured using terminal deoxynucleoside transferase dUTP nick end labeling (TUNEL) assay (11684795910, Roche Applied Science, Germany) according to the manufacturer’s protocol. The nuclei were counterstained with Hoechst 33342 (H3570, Thermo Fisher, United States). The stained cells were visualized using a laser scanning confocal microscope (LSM510, Carl Zeiss, Germany) at the 488 nm laser excitation and the emission in the range of 515–565 nm for TUNEL signal. The percentage of TUNEL-positive cells was determined by counting 200–250 cells in 5 randomly selected fields.

### Statistical Analyses

Data are presented as mean ± SEM. Differences between means were determined using the One-way ANOVA for group-paired observations. Differences were considered statistically significant when *p* < 0.05.

## Results

### PNS Attenuated Mitochondrial Injury and ROS Accumulation in Cardiac Myocytes With TG-Induced ER Stress

**FIGURE 1:**
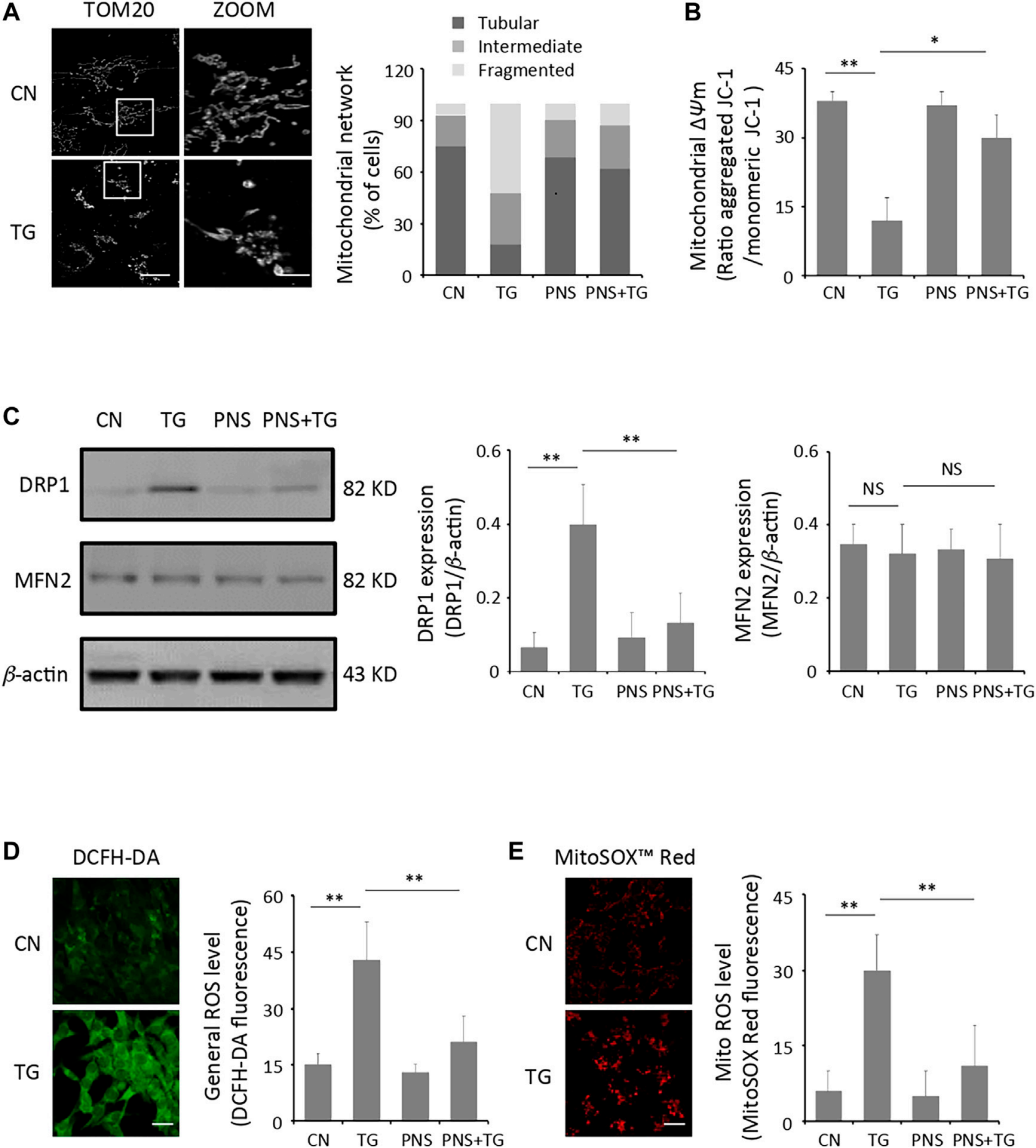
PNS prevents TG-induced mitochondria injury and ROS accumulation. **(A)** Primary cultured cardiomyocytes, either untreated (CN group) or pretreated with 40 μg/ml PNS for 12 h (PNS group), before addition of 1 μM thapsigargin (TG group or PNS plus TG group) for 12 h were immunofluorescenced with the primary anti-TOM20 antibody and imaged by a laser scanning confocal microscopy. Representative images in CN and TG group. Scale bar: 30 μm; in box: 10 μm. Bar graph shows percentage of cardiomyocytes with tubular, intermediate or fragmented mitochondria in various groups as indicated (80–100 cells). **(B)** Primary cultured cardiomyocytes treated as in A were stained with JC-1 and analyzed by a plate reader. Bar graph shows the ratio of aggregated JC-1 (red)/monomeric JC-1 (green) as the mitochondrial membrane potential (Δ*ψ*m) (Mean ± SEM; 80–100 cells; *p < 0.05, **p < 0.01 relative to CN group, or indicated group). **(C)** Primary cultured cardiomyocytes treated as in A were immunoblotted with the antibodies to DRP1, MFN2 and *β*-actin. Bands were quantified relative to *β*-actin by densitometry (Mean ± SEM; **p < 0.01 relative to CN group, or indicated group). **(D)** Primary cultured cardiomyocytes treated as in A were stained with DCFH-DA and imaged by a laser scanning confocal microscopy. Representative images in CN and TG group. Scale bar: 80 μm. Bar graph shows the fluorescence intensity of DCFH-DA as the general ROS level (Mean ± SEM; 80–100 cells; **p < 0.01 relative to CN group, or indicated group). **(E)** Primary cultured cardiomyocytes treated as in A were stained with MitoSOX™ Red and imaged by a laser scanning confocal microscopy. Representative images in CN and TG group. Scale bar: 80 μm. Bar graph shows the fluorescence intensity of MitoSOX Red as the mitochondrial ROS level (Mean ± SEM; 80–100 cells; **p < 0.01 relative to CN group, or indicated group).

ER and mitochondria are closely connected organelles within cells. ER stress contributes to mitochondrial dysfunction, which is a key factor to increase cardiac injury (Chen et al., 2017). Therefore, we first examined the mitochondrial structure in cardiomyocytes in the presence or absence of TG-induced ER stress. Confocal images showed that most of mitochondria appeared as long, tubular or branched structures that spread throughout the entire cytoplasm in the untreated cells. Quantification of mitochondrial morphology showed the percentage of cells exhibiting tubular, intermediate and fragmented mitochondria structures. Both the images and the graph showed that the arrangement of mitochondria in TG-induced cardiomyocytes is disorganized and fragmented, and a significant decreased number of tubular-like mitochondria displayed in TG-induced cardiomyocytes (Figure 1A). We then performed the immunoblot analyses against proteins that involved in mitochondrial fission (DRP1) and fusion (MFN2), which contributes to better illustrating the effect of TG-induced ER stress on the regulation of the mitochondria morphology. DRP1 expression in the untreated cells was revealed 3-fold higher than that in TG-induced cardiomyocyte (*p* < 0.01), whereas there was no significant difference of the MFN2 expression in both the untreated and TG-induced cells (Figure 1C).

Mitochondrial damage is often accompanied by a decrease in mitochondrial membrane potential (Δ*ψ*m) as well as ROS accumulation (Thangaraj et al., 2020). Live cell measurements of Δ*ψ*m using the fluorescence indicator JC-1 revealed large decreases of fluorescence in cardiomyocytes with TG-induced ER stress compared to that in the untreated cells (*p* < 0.01), suggesting a large amount of mitochondria depolarization induced by TG (Figure 1B). In addition, the intracellular general ROS and mitochondrial ROS were measured using the fluorescent indicators DCFH-DA and MitoSOX™ Red, respectively. Cardiomyocytes produced baseline levels of the general and the mitochondrial ROS whereas TG stimulation increased both the general ROS generation and the mitochondrial ROS production significantly (2-3 folds, *p* < 0.01) compared to those in the untreated cells (Figure 1D,E).

PNS has been illustrated can ameliorate mitochondrial injury induced by an anti-cancer drug-cisplatin (Li et al., 2020). We then investigated the effect of PNS on TG-induced mitochondrial injury. Interestingly, pretreatment of PNS relieved disorganization and fragmentation of the mitochondria induced by TG, and the elevation of DRP1 expression was downregulated by PNS pretreatment in TG-induced cells. In addition, the depolarization of mitochondria membrane, as well as the accumulation of the general and the mitochondrial ROS induced by TG were prevented by PNS pretreatment.

### PNS Promoted Autophagy in Cardiac Myocytes With TG-Induced ER Stress

**FIGURE 2:**
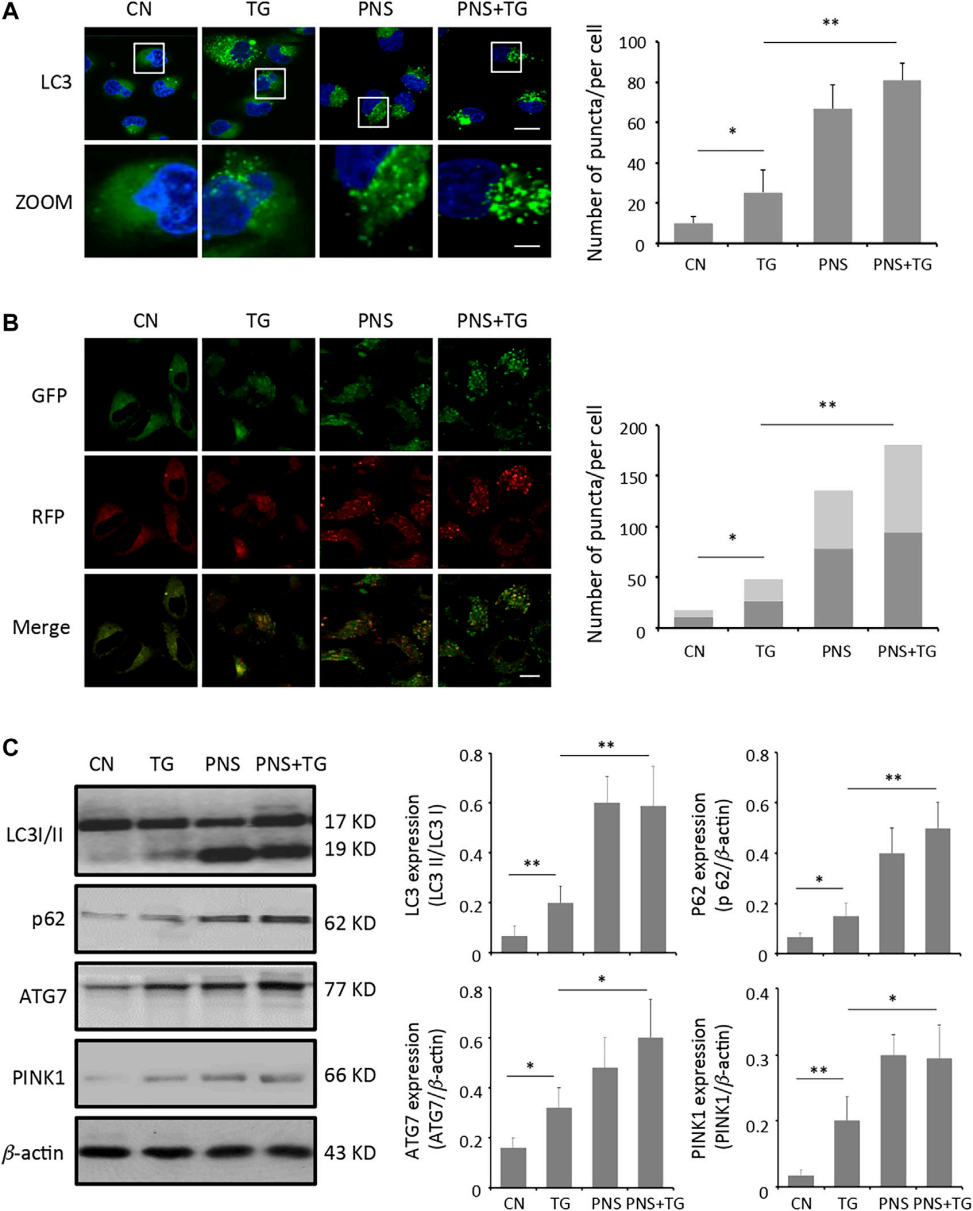
PNS promotes autophagy and autophagic flux. **(A)** Primary cultured cardiomyocytes, either untreated (CN group) or pretreated with 40 μg/ml PNS for 12 h (PNS group), before addition of 1 μM thapsigargin (TG group or PNS plus TG group) for 12 h were immunofluorescenced with the primary anti-LC3B antibody and imaged by a laser scanning confocal microscopy. Scale bar: 30 μm; in box: 10 μm. Bar graph shows the number of LC3 puncta per cell in various groups as indicated (Mean ± SEM; 80–100 cells; *p < 0.05, **p < 0.01 relative to CN group, or indicated group). **(B)** Primary cultured cardiomyocytes treated as in A were transfected with mRFP-GFP-LC3 and imaged by a laser scanning confocal microscopy. Scale bar: 30 μm. Bar graph shows the number of red only and yellow LC3 puncta per cell in various groups as indicated (Mean ± SEM; 60–80 cells; *p < 0.05, **p < 0.01 relative to CN group, or indicated group). **(C)** Primary cultured cardiomyocytes treated as in A were immunoblotted with the antibodies to LC3B, p62, ATG7, PINK1 and *β*-actin. Bands were quantified relative to *β*-actin or proteins as indicated by densitometry (Mean ± SEM; *p < 0.05, **p < 0.01 relative to CN group, or indicated group).

Evidence has shown that PNS may affect autophagy and is involved in autophagy signaling pathways (Liu et al., 2019b). To examine the effect of PNS on occurrence of autophagy in cardiomyocytes in the presence and absence of TG stimulation, LC3B antibody immunofluorescence labeling was adopted. Quantitation of the fluorescent images demonstrated that the number of LC3 antibody labeled puncta in PNS-pretreated cardiomyocytes was significantly increased by 2-fold compared to that in the untreated cells (*p* < 0.01), and it was further enhanced in the PNS pretreated cells with TG exposure by 3-fold as compared to that in the untreated cardiomyocytes or TG-stimulated cardiomyocytes (*p* < 0.01) (Figure 2A).

Autophagic flux was measured using the autophagy tandem sensor mRFP-GFP-LC3. Autophagosomes were observed as yellow puncta and autolysosomes appeared as red only puncta in the merged images. Autophagic flux was determined based on the increase in the percentage of the red only spots in the merged images. Compared with the untreated cells, both the percentage of red puncta and yellow puncta significantly increased in PNS-pretreated cells or PNS-pretreated cells with TG stimulation (*p* < 0.01), indicating that PNS-pretreatment promoted high level of autophagy occurrence and high autophagic flux in cardiomyocytes (Figure 2B).

Immunoblot analyses of TG-induced cardiomyocytes against autophagy markers were applied to further identify the effect of PNS on autophagy in cardiomyocytes. The light-chain 3-cleavage ratio (LC3 II/I) was 2.5 folds higher in PNS-pretreated cells compared to that in cells absent of PNS pretreatment (*p* < 0.01). The level of the autophagosome scaffolding protein p62 was 2–2.5 folds higher in PNS-pretreated cells compared to that in cells without PNS pretreatment (*p* < 0.01). The expression of the E1 enzyme for the ubiquitin-like autophagy protein (ATG7) was 1.5-2 folds higher in PNS-pretreated cells in comparison to that in cells absent of PNS pretreatment (*p* < 0.05). In addition, consistent with the increased level of autophagy, the ubiquitin-mediated mitophagy kinase, PINK1 expression was increased 1.5-fold in PNS pretreated cardiomyocytes either with or without TG stimulation relative to that in cardiomyocytes without PNS pretreatment, suggesting that PNS may induce mitophagy in cardiomyocytes (*p* < 0.05) (Figure 2C).

### Inhibition of Autophagy Diminished PNS Prevention of TG-Induced Mitochondrial Injury, ROS Accumulation and Calcium Dyshomeostasis

**FIGURE 3:**
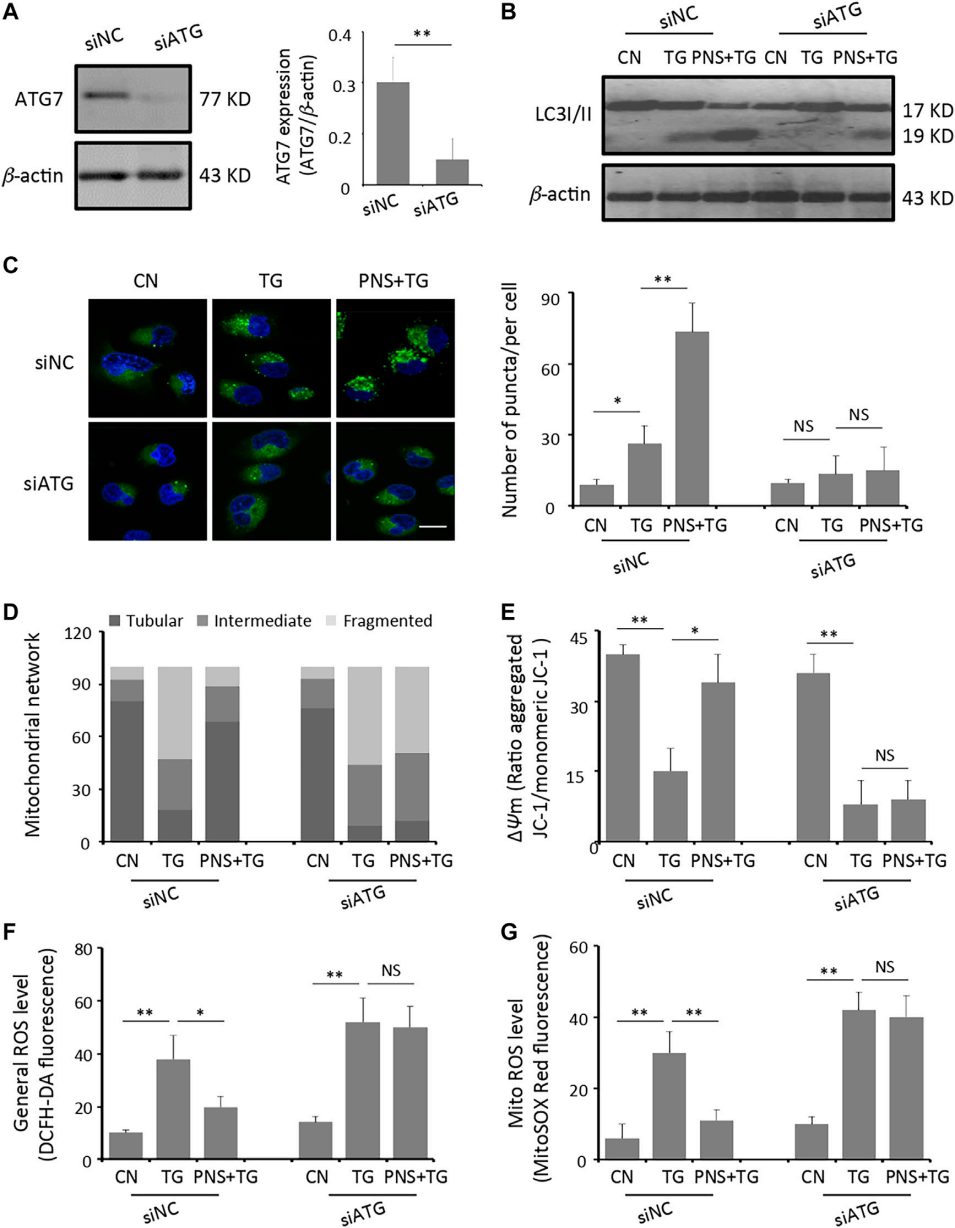
Inhibition of autophagy diminishes PNS prevention of TG-induced mitochondria injury and ROS accumulation. **(A)** SiRNA negative control (siNC group) or siATG7 (siATG7 group) transfected H9c2 cells were identified by immunoblotting of the anti-ATG7 and *β*-actin antibodies. Bands were quantified relative to *β*-actin by densitometry (Mean ± SEM; **p < 0.01 relative to siNC group). **(B)** SiNC or siATG7 transfected H9c2 cells, either untreated (CN group) or pretreated with 40 μg/ml PNS for 12 h before addition of 1 μM TG (TG group or PNS plus TG group) for 12 h were immunoblotted to the antibodies to LC3B and *β*-actin **(C)** SiNC or siATG7 transfected H9c2 cells, treated as in B, were immunofluorescenced with the primary anti-LC3B antibody and imaged by a laser scanning confocal microscopy. Scale bar: 30 μm. Bar graph shows the number of LC3 puncta per cell in various groups as indicated (Mean ± SEM; 80–100 cells; *p < 0.05, **p < 0.01 relative to CN group, or indicated group). **(D)** SiNC or siATG7 transfected H9c2 cells, treated as in B, were immunofluorescenced with the primary anti-TOM20 antibody. Bar graph shows the percentage of cells with tubular, intermediate or fragmented mitochondria in various groups as indicated (80–100 cells) **(E)** SiNC or siATG7 transfected H9c2 cells, treated as in B, were stained with JC-1 and analyzed by a plate reader. Bar graph shows the ratio of aggregated JC-1 (red)/monomeric JC-1 (green) as the mitochondrial membrane potential (Δ*ψ*m) (Mean ± SEM; 80–100 cells; *p < 0.05, **p < 0.01 relative to CN group, or indicated group). **(F)** SiNC or siATG7 transfected H9c2 cells, treated as in B, were stained with DCFH-DA. Bar graph shows the fluorescence intensity of DCFH-DA as the general ROS level. (Mean ± SEM; 80–100 cells; *p < 0.05, **p < 0.01 relative to CN group, or indicated group). **(G)** SiNC or siATG7 transfected H9c2 cells, treated as in B, were stained with MitoSOX™ Red. Bar graph shows the fluorescence intensity of MitoSOX Red as the mitochondrial ROS level (Mean ± SEM; 80–100 cells; **p < 0.01 relative to CN group, or indicated group).

To further determine the effect of autophagy on PNS prevention of TG-induced mitochondrial injury, ROS accumulation and calcium dyshomeostasis, we next generated H9c2 cells with the genetic ablation of autophagy using siRNA against the essential autophagy enzyme ATG7 (siATG7). Transient transfection of H9c2 cells with siRNA-ATG7 reduced the expression of ATG7 by 67% (Figure 3A). Immunoblot and immunofluorescence of the LC3B antibody found that significant lower autophagy levels (identified by the LC3II/I ratio and the amount of LC3 puncta) were exhibited in siATG7 transfected cells than that in siNC transfected cells with PNS pretreatment in the presence or absence of TG stimulation (Figure 3B,C). Interestingly, PNS can no longer reverse the alterations of mitochondrial network disruption, depolarization of mitochondrial membrane as well as accumulation of the general and the mitochondrial ROS production triggered by TG with the suppression of autophagy (Figure 3D–G).

**FIGURE 4:**
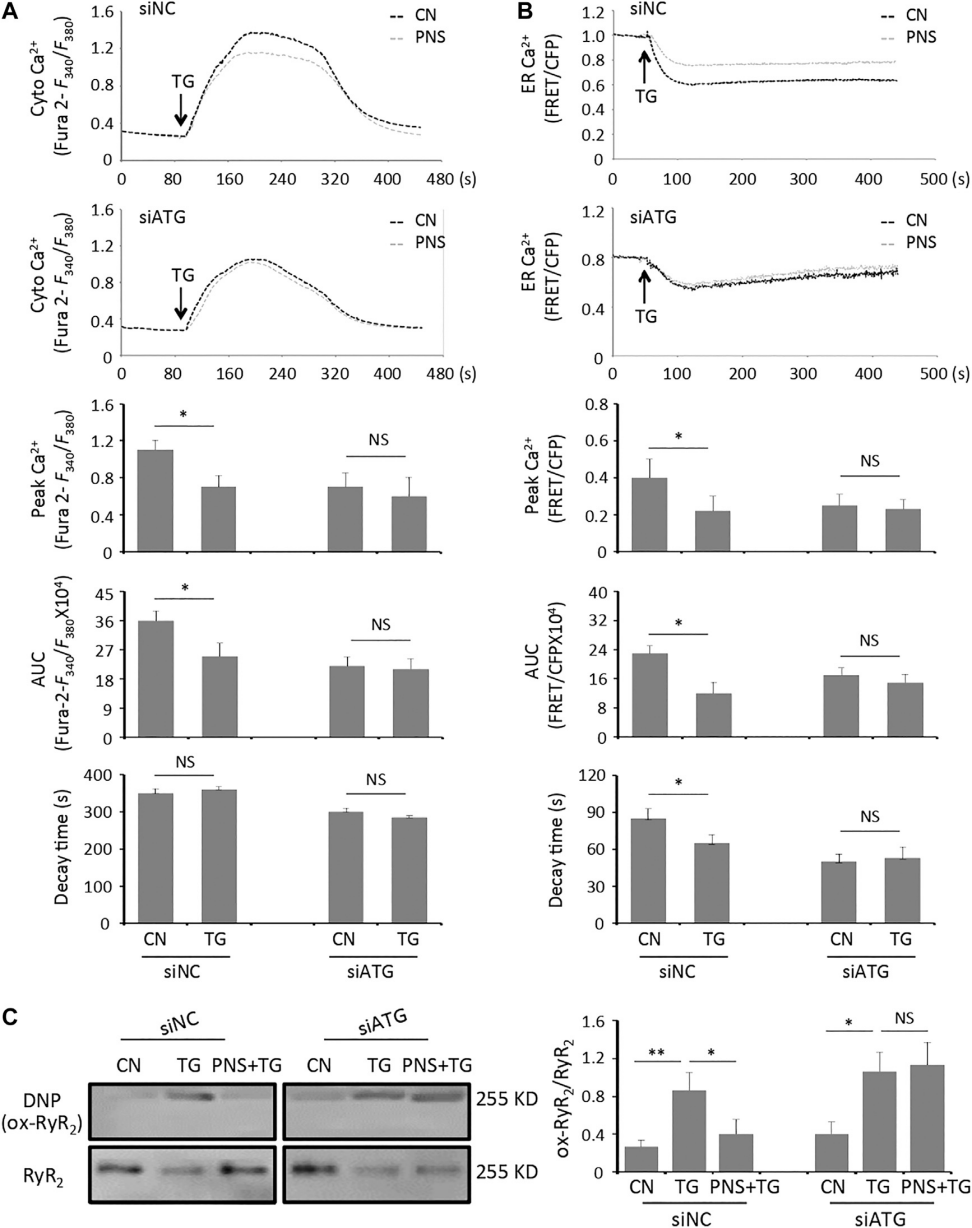
Inhibition of autophagy diminishes PNS suppression on cytosolic Ca^2+^ transits and ER Ca^2+^ releases, as well as RyR_2_ oxidation evoked by TG. **(A)** Representative recordings of TG-evoked cytosolic Ca^2+^ dynamics were recorded by Fura-2 ratios (*F*_340_/*F*_380_) in siNC (upper panel) or siATG7 (bottom panel) transfected H9c2 cells with (gray, PNS group) or without (dark, CN group) PNS pretreatment (40 μg/ml; 12 h), as indicated. Bar graphs show the cytosolic Ca^2+^ peak amplitude, the area under curve (AUC), as well as the time decay of Ca^2+^ transits in response to TG stimulation (Mean ± SEM; 50–60 responding cells; *p < 0.05 relative to CN group in siNC or siATG7 transfected cells). **(B)** Representative recordings of TG-induced ER Ca^2+^ dynamics were recorded by the FRET-to-CFP emission ratio (FRET/CFP) in siNC (upper panel) or siATG7 (bottom panel) transfected H9c2 cells with (gray, PNS group) or without (dark, CN group) PNS pretreatment (40 μg/ml; 12 h), as indicated. Bar graphs show the ER Ca^2+^ peak amplitude, the area under curve (AUC), as well as the time decay of Ca^2+^ release in response to TG stimulation (Mean ± SEM; 20–30 responding cells; *p < 0.05 relative to CN group in siNC or siATG7 transfected cells). **(C)** SiNC or siATG7 transfected H9c2 cells, either untreated (CN group) or pretreated with 40 μg/ml PNS for 12 h before addition of 1 μM TG (TG group or PNS plus TG group) for 12 h were immunoblotted with the antibodies to DNP and RyR_2_. Bands were quantified relative to RyR_2_ by densitometry (Mean ± SEM; *p < 0.05, **p < 0.01 relative to CN group, or indicated group).

To further evaluate the functional effects of inhibition autophagy on PNS mediated Ca^2+^ handling, we performed calcium imaging on the intracellular cytosolic Ca^2+^ transit and ER Ca^2+^ release evoked by TG in siATG7 and siNC transfected cells in the presence and absence of PNS pretreatment (Figure 4 A,B). Both cytosolic Ca^2+^ and ER Ca^2+^ dynamics showed that TG-induced the elevation of cytosolic Ca^2+^ transit and ER Ca^2+^ release was significantly reduced by PNS pretreatment in siNC transfected cells (Figure 4A,B, peak amplitude and area under the curve (AUC)). However, in siATG7 transfected cells, no significant differences in both the peak amplitude and AUC of the cytosolic Ca^2+^ and the ER Ca^2+^ dynamics were detected in the presence and absence of PNS pretreatment, suggesting that PNS pretreatment did not affect the Ca^2+^ handling with the inhibition of autophagy. These results indicated that PNS pretreatment reduces the intracellular cytosolic Ca^2+^ transit and ER Ca^2+^ release upon TG stimulation was related to autophagy.

Previous study has reported that PNS decreases RyR_2_ expression and regulates the intracellular Ca^2+^ dynamics through down-regulation of RyR_2_ expression (Chen et al., 2019). We then assessed the effect of PNS on the alterations of RyR_2_ oxidation in siNC and siATG7 transfected cells with TG stimulation. In the group of siNC transfected cells, the level of densitometry showed a 2-fold increase of the NDP signal (Ox-RyR_2_) in TG-stimulated cells, indicating that TG increases the oxidation level of RyR_2_ (*p* < 0.01), whereas the oxidation level of RyR_2_ was significantly decreased in PNS-pretreated cells with TG stimulation compared to that in TG alone treated cells (*p* < 0.05). However, in the group of siATG7-transfected cells, there were no differences in the NDP (Ox-RyR_2_) signal in TG-induced cells either with or without PNS pretreatment (Figure 4C), suggesting that PNS suppression on TG-induced RyR_2_ oxidation was related to autophagy.

### Inhibition of Autophagy Abolished the Effect of PNS Protection Against TG-Induced ER Stress Response and Associated Apoptosis

**FIGURE 5:**
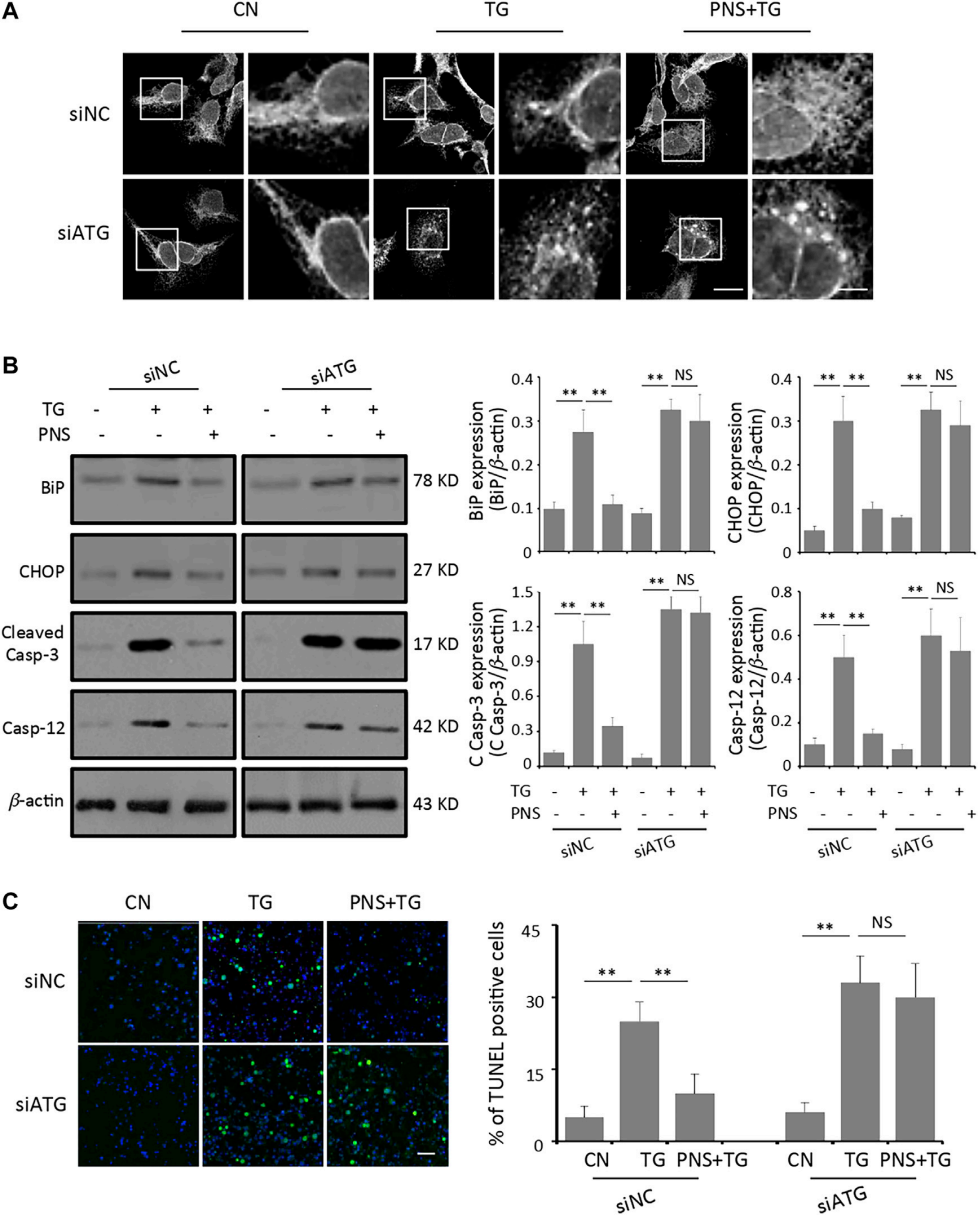
Inhibition of autophagy abolishes PNS protection on TG-induced ER stress response and associated apoptosis. **(A)** SiNC or siATG7 transfected H9c2 cells, either untreated (CN group) or pretreated with 40 μg/ml PNS for 12 h before addition of 1 μM TG (TG group or PNS plus TG group) for 12 h were immunofluorescenced with the primary anti-calnexin antibody and imaged by a laser scanning confocal microscopy. Scale bar: 30 μm; in box: 10 μm. **(B)** SiNC or siATG7 transfected H9c2 cells, treated as in A, were immunoblotted with the antibodies to BiP, CHOP, Cleaved Caspase-3 and Caspase-12 as well as *β*-actin. Bands were quantified relative to *β*-actin by densitometry (Mean ± SEM; **p < 0.01 relative to CN group, or indicated group). **(C)** SiNC or siATG7 transfected H9c2 cells, treated as in A, were stained with TUNEL and imaged by a laser scanning confocal microscopy. Scale bar: 200 μm. Bar graph shows the percentage of TUNEL positive cells (Mean ± SEM; 200–250 cells; **p < 0.01 relative to CN group, or indicated group).

To further determine the effect of autophagy on PNS protection upon TG-induced ER stress response and associated apoptosis, the ER network structure as well as the levels of ER stress-related proteins-BiP and CHOP, together with the apoptotic genes-Cleaved Caspase-3 and Caspase-12 were detected by immunofluorescence and immunoblots, respectively. Similar to the previous data (Chen et al., 2019), PNS exerted significant effects on TG-induced disruption of ER network and up-regulation of BiP, CHOP, Cleaved Caspase-3 and Caspase-12 expressions in siNC transfected cells (Figure 5 A and B). However, in siATG7 transfected cells, the disruption and condensation of the ER tubular network induced by TG were no longer affected by PNS pretreatment (Figure 5A). In addition, there were no significant difference in the expressions of BiP, CHOP, Cleaved Caspase-3 and Caspase-12 in TG-induced cells in the presence or absence of PNS pretreatment (Figure 5B). In addition, the cell viability assay using TUNEL staining also showed that the high number of TG-induced apoptotic cells was significantly attenuated by PNS pretreatment in siNC transfected cell but that was no longer altered by PNS pretreatment in TG-induced siATG7 transfected cells (Figure 5C). These results suggested that PNS protection of ER stress response and associated cell apoptosis was mediated by autophagy.

In summary, PNS pretreatment prevents cardiac myocytes from TG-induced ER stress, mitochondria injury and associated apoptosis. Furthermore, inhibition of autophagy rendered cells vulnerable to TG-induced mitochondria damage, ER stress and associated apoptosis with PNS pretreatment suggests that autophagy plays important roles in PNS promotion of cell survival toward ER stress. Our results supported the statement that autophagy is important in maintaining cellular longevity and the consequent functional integrity, and therefore, could be a new potential target of pharmaceutical management of PNS on the cardiovascular diseases.

## Discussion

Autophagy plays an essential role in cell survival under certain stress conditions such as nutrient or growth factor deprivation, ER stress, hypoxia, oxidative stress, DNA damage, protein aggregates and damaged organelles (Kroemer et al., 2010). Our previous study has shown that PNS can protect cardiac myocytes against ER stress response and associated cell death (Chen et al., 2019). Accumulating evidence shows that PNS and its main components are involved in autophagy pathway and thus promoting cell survival (Liu et al., 2019a; Liu et al., 2019b). However, the interaction between ER stress and autophagy pathways, in particular, whether PNS protection against ER stress response and associated cell death is related to autophagy still remains unknown.

In this study, we have demonstrated that PNS protection against ER stress and associated cell death in cardiac myocytes is related to prevention of ROS production and promotion of autophagy, and thus inhibition of autophagy abolishes the protective effect of PNS on ER stress response and associated cell apoptosis by the following evidence: Firstly, PNS protects against TG-induced mitochondrial injury and ROS accumulation in cardiac myocytes. Secondly, PNS enhances autophagy in cardiac myocytes with TG-induced ER stress. Thirdly, inhibition of autophagy diminishes PNS prevention of TG-induced mitochondrial injury, ROS accumulation and disruption of Ca^2+^ homeostasis. Last but not least, inhibition of autophagy abolishes PNS protection against TG-induced ER stress response and associated apoptosis.

Myocardium is a highly oxidative tissue. Mitochondrial fusion, fission, biogenesis and mitophagy have been implicated in cardiovascular disease and play crucial roles in maintaining optimal heart performance (Vásquez-Trincado et al., 2016). Given the unique and highly dynamic configuration of mitochondria in cardiomyocytes, the effects of PNS on TG-induced mitochondrial fragmentation, membrane depolarization and ROS over-production are investigated. ER stress is accompanied with an increase in intracellular Ca^2+^ level and rising of the intracellular ROS that mediated the mitochondrial permeability transition pore (mPTP) opening, and cardiomyocytes apoptosis and necrosis (Zhu et al., 2018). Acute-induced ER stress increased the activity of the UPR, leading to dysregulated mitochondrial function (Poirier et al., 2019). We found that TG interrupts mitochondria network, stimulates the mitochondrial membrane depolarization and induces the intracellular and mitochondrial ROS accumulation, however, pretreated PNS mitigated mitochondrial injury and ROS accumulation induced by TG (Figure 1). We further found that PNS pretreatment can increase the formation of autophagic vacuoles and the autophagic influx, as well as up-regulate the expression of autophagy-related proteins (Figure 2), which is consistent with the previous investigation that PNS attenuates myocardial ischemia-reperfusion injury by decreasing in the myocardial tissue superoxide dismutase, ROS, the attenuation of mitochondrial damages of myocardial cells, and the increase of mitochondria autophagosome in myocardial cells that mediated by the HIF-1α/BNIP3 autophagy pathway (Liu et al., 2019b). Notably, PNS was also demonstrated to protect from cisplatin-induced mitochondrial injury by enhancing hypoxia-inducible factor-1α (HIF-1α)-mediated mitochondrial autophagy. Cisplatin induced mitochondria injury and ROS overproduction, which in turn damaged the mitochondria. Interestingly, PNS enhanced HIF-1α-mediated mitochondrial autophagy to selectively remove damaged mitochondria, which contributes to a reduction of ROS production (Li et al., 2020). To reveal the relationship between autophagy and ER stress response and associated apoptosis on cardiac myocytes with PNS pretreatment, we further transfected cells with siATG7 to inhibit autophagy. Interestingly, suppression of autophagy eliminates the protective effects of PNS on mitochondrial injury, ROS elevation as well as mitochondrial membrane depolarization (Figure 3), suggesting that PNS protection on ER stress-induced mitochondrial injury is related to the promotion of autophagic flux and the induction of selective autophagy, which is in line with the previous report that autophagy pathways play important role in amelioration of oxidative stress-mediated mitochondrial dysfunction and ER stress (Tang et al., 2017). Increasing evidence has shown that ER stresses, oxidative stress and autophagy are closely related. Both oxidative stress and ER stress are involved in palmitic acid-induced H9c2 cell apoptosis (Yang et al., 2019). ER stress and autophagy are also found to be involved in H9c2 cells during hypoxia/reoxygenation (H/R) injury (Guan et al., 2019). Our data also have suggested that PNS protection against ER stress and ROS accumulation is closely related to autophagy. TG inhibits Ca^2+^ reuptake from ER and induces overload of the cytosolic and mitochondrial Ca^2+^ that results in mitochondrial injury, ROS release and activation of the apoptotic pathways subsequently. PNS enhances autophagy to selectively remove damaged mitochondria and reduce ROS overproduction.

In the past years, mounting evidences have shown that calcium signaling is involved in apoptotic cell death caused by certain stimulation (Zhivotovsky and Orrenius, 2011). TG-induced injury and permeation of mitochondria within cardiac myocytes manifest increased ability to accumulate intracellular Ca^2+^ (Sheng et al., 2018). And also TG inhibited Ca^2+^ reuptake through sarcoendoplasmic reticulum (SR) calcium transport ATPase (SERCA) pump to recycle the intracellular Ca^2+^ accumulation (Sehgal et al., 2017). Our previous study has shown that PNS pretreatment reversed the elevation of cytosolic Ca^2+^ overload, ER Ca^2+^ release, and mitochondrial Ca^2+^ uptake induced by TG via down regulating the expression of RyR_2_ (Chen et al., 2019). And the current study has shown that PNS decreases TG-induced intracellular ROS accumulation and promotes autophagy (Figures 1, 2). Therefore, it is important to elucidate whether autophagy is involved in PNS prevention of intracellular Ca^2+^ dyshomeostasis as well as RyR_2_ oxidation in response to TG stimulation. We found that the effect of PNS on TG-induced cytosolic Ca^2+^ transit as well as ER Ca^2+^ release is suppressed by inhibition of autophagy. In addition, densitometry showed that blocking autophagy increases the TG-induced oxidation of RyR_2_ (Figure 4). These data are consistent with the other investigations, suggesting that mito-ROS oxidizes the RyR_2_, leading to increased SR Ca^2+^ leak and generation of spontaneous Ca^2+^ waves (SCWs) (Kim et al., 2017; Murphy et al., 2019). Targeted enhancement of autophagy effectively stabilized RyR_2_-mediated Ca^2+^ release via down-regulation of mito-ROS production (Hamilton et al., 2020).

To further verify whether autophagy plays a role in PNS protection against ER stress response and associated cell death, we examined the effects of PNS on TG-induced ER network morphology, expressions of ER stress markers (BiP and CHOP) and cell apoptotic markers (Caspase-3 and Caspase-12), as well as the cell viability in the presence and absence of autophagy inhibition. Our data indicated that inhibition of autophagy eliminates the protective effects of PNS against ER stress response and associated cell death (Figure 5), which is consistent with the report that under ER stress conditions, autophagy can be activated in order to undertake the degradative machinery to attenuate the ER stress. Inhibition of autophagy by trehalose exacerbates cell damage, and failed to exert neuroprotective function (Halbe and Rami, 2019). Interestingly, we also found that PNS down-regulates the expressions of CHOP and Caspase-12, suggesting that CHOP-mediated ER stress apoptosis pathway may be involved in PNS protection against cardiac myocytes cell apoptosis, which is consistent with the previous report that saponins of Panax notoginseng suppressed the palmitate-induced ROS generation, and the ER stress-associated eIF2α/ATF4/CHOP and Caspase-12 pathways in RGC-5 cells (Wang et al., 2016). GR1, one of the main components of PNS, also has been found to prevent cardiomyocyte apoptosis and delay the onset of ER stress by inhibiting the expression of pro-apoptosis proteins CHOP, Caspase-12, and P-JNK (Yu et al., 2016).

**FIGURE 6:**
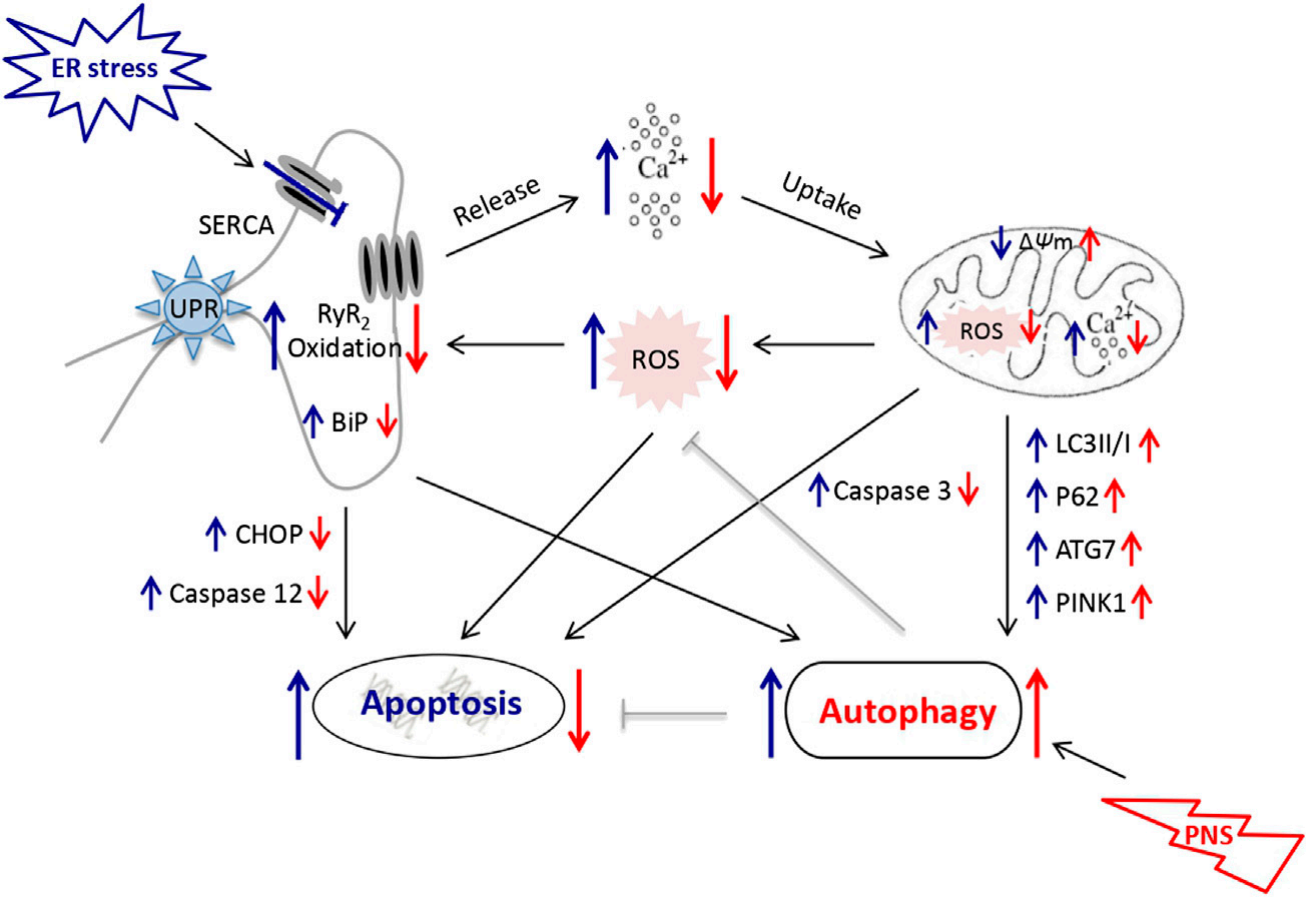
Schematic diagram depicting PNS protection against TG-induced mitochondrial injury, ER stress and associated cell apoptosis. TG inhibits Ca^2+^ reuptake from ER and induces overload of the cytosolic and mitochondrial Ca^2+^ that results in mitochondrial injury, ROS release and activation of the apoptotic pathways subsequently. PNS enhances autophagy to selectively remove damaged mitochondria and results in reduced ROS production and thus the oxidation of RyR_2_, which in turn inhibits Ca^2+^ release from ER. PNS thereby attenuates TG-induced Ca^2+^-dependent ER stress and alleviates the associated apoptosis.

Taken together, the underlying mechanism of PNS protection against ER stress and associated apoptosis via mitochondria/autophagy pathways might be that TG exposure induces Ca^2+^-dependent ER stress and leads to mitochondrial injury. The accumulation of fragmented and damaged mitochondria subsequently triggers ROS overproduction and activation of the mitochondrial apoptotic pathway. However, PNS restores autophagic flux and promotes autophagy that selectively eliminates damaged mitochondria and misfolded proteins to maintain mitochondrial quality and alleviate ROS production. PNS attenuates RyR_2_ oxidation mediated by mitochondria/ROS/autophagy pathway results in the inhibition of Ca^2+^ release from ER, which in turn attenuates Ca^2+^-dependent ER stress, and thereby alleviates ER stress-associated cell apoptosis (Figure 6).

## Conclusion

This study demonstrates that PNS protection against ER stress response and associated cell death is related to its effects on ROS production and autophagy, suggesting that PNS could attenuate cardinal injury through the interaction of ER stress and autophagy pathways. These data provide new insights for cellular and molecular mechanisms of the herbal medicine–PNS as a potential preventive approach to manage the cardiovascular diseases.

## Data Availability

The raw data supporting the conclusions of this article will be made available by the authors, without undue reservation, to any qualified researcher.

## Abbreviations:

PNS, Panax notoginseng saponins; ER, endoplasmic reticulum; HPLC, high performance liquid chromatograph; TG, thapsigargin; UPR, unfolded protein response; BiP, immunoglobulin heavy chain-binding protein; CHOP, C/EBP homologous protein; RyR_2_: ryanodine receptor type-2; AUC, area under the curve; FRET, fluorescence resonance energy transfer; ROS, reactive oxygen species; SiRNA, small interfering RNA; LC3, anti-microtubule-associated protein 1A/1B-light chain 3; Δψm: mitochondrial membrane potential; TUNEL, terminal deoxynucleotidyl transferase dUTP nick end labeling.
